# Morphological, Immunocytochemical, and Biochemical Studies of Rat Costal Chondrocytes Exposed to IL-1*β* and TGF-*β*1

**DOI:** 10.1155/2017/9747264

**Published:** 2017-07-04

**Authors:** Xiaoli Li, Xiaoyong Ren, Sisi Li, Jianmin Liang, Xiaoyan Zhao, Ting Wang, Zhenghui Wang

**Affiliations:** ^1^Department of Dermatology, The Second Affiliated Hospital, Xi'an Jiaotong University, Xi'an 710004, China; ^2^Department of Otolaryngology-Head and Neck Surgery, The Second Affiliated Hospital, Xi'an Jiaotong University, Xi'an 710004, China; ^3^Department of Medicine, Beth Israel Deaconess Medical Center, Harvard Medical School, Boston, MA, USA

## Abstract

This study was undertaken to determine the effects of IL-1*β* and TGF-*β*1 on the expression of differentiation-associated genes in chondrocytes in vitro. Rat costal chondrocytes were exposed to different concentrations of IL-1*β* and TGF-*β*1 for 48 h and tested for gene expression. IL-1*β* increased the expression of aggrecanase-1 and aggrecanase-2 and decreased the content of aggrecan and collagen II. Low concentration of TGF-*β*1 decreased the expression of aggrecan and collagen II and increased the expression of aggrecanase-2. However, the level of aggrecanase-1 was significantly elevated in the presence of high concentration of TGF-*β*1. IL-1*β* and TGF-*β*1 show the ability to modulate the production of aggrecan and collagen II in chondrocytes in vitro.

## 1. Introduction

Cartilage regeneration is often needed in orthopedic or plastic surgery for the repair of cartilaginous defects. However, due to the limited regenerative capacity of the cartilage tissue, the treatment of various cartilaginous lesions remains a challenge to clinicians. Recently, tissue engineering has emerged as a new method in which a combination of cells, scaffold, and bioactive agents is used to fabricate functional new tissue to replace damaged cartilage [1–3]. Chondrocytes seeded onto appropriate scaffolds can produce cartilage-like tissues, thus representing a major cell source for cartilage tissue engineering [4].

Once chondrocytes had been successfully cultured in vitro, study of cartilage matrix progressed rapidly. However, some issues remain unresolved, such as the dedifferentiation which occurs in cultured chondrocytes in vitro. As the number of passages increases, the cells undergo a change in phenotype and the morphology becomes more fibroblast-like [5]. Dedifferentiated chondrocytes lose their ability to form a matrix; they synthesize predominantly type-1 collagen. There is an associated downregulation of both aggrecan and type II collagen, the major protein produced by chondrocytes in cartilage [6, 7]. Thus, maintenance of the chondrocyte phenotype during a prolonged monolayer culture, delaying dedifferentiation, is crucial to chondrocyte transplantation and tissue engineering.

Many studies suggested that interleukin-1beta (IL-1*β*) and transforming growth factor beta 1 (TGF-*β*1) are implicated in cell differentiation [8, 9]. However, their roles in chondrocyte differentiation are not completely understood. In this study, we examined the effects of different concentrations of IL-1*β* and TGF-*β*1 on the production of collagen II, aggrecanase-1, aggrecanase-2, and aggrecan in chondrocytes, in order to explain the mechanism of IL-1*β* and TGF-*β*1 on chondrocyte differentiation.

## 2. Materials and Methods

### 2.1. Materials

Female Sprague-Dawley rats weighing about 120 g were purchased from the Laboratory Animal Center of Xi'an Jiaotong University (Xi'an, China). DMEM and fetale bovine serum (FBS) were purchased from Invitrogen. Hyaluronidase, collagenase II, trypsin, and alcian blue 8GX (Sigma), mouse anti-rat collagen II monoclonal antibody (Neomarker), goat anti-rat aggrecan monoclonal antibody (Santa Cruz Biotechnology), DAB kit (Beijing Zhongshan), RNA isolation kit (Shanghai Feijie Company), and First Strand CDNA Synthesis Kit (MBI Fermentas) are the materials used.

### 2.2. Isolation and Culture of Chondrocytes

Isolation and culture of rat costal chondrocytes were performed as described previously [10]. In brief, rats were killed by cervical dislocation and the ribs were removed and the cartilage was collected and cut into 1 mm^3^ pieces. The tissue samples were digested with hyaluronidase and collagenase II, and the isolated cells were cultured at 37°C under a humidified atmosphere of 5% CO_2_. Cells were subcultured at a ratio of 1 : 1 after treatment with 0.25% trypsin/EDTA. The isolated chondrocytes were identified by immunostaining for collagen II and aggrecan. In this study, cells at passage 2 were used.

### 2.3. Treatment of Chondrocytes with IL-1*β* and TGF-*β*1

Cells (5 × 10^4^) were seeded in triplicates onto 24-well plates and incubated with different concentrations of IL-1*β* or TGF-*β*1 (1, 10, and 100 ng/ml) for 48 h. The cells were then collected and tested for gene expression.

### 2.4. Immunocytochemical Analysis

Cells were plated on cover slips and allowed to grow to 80% confluence. Cells were washed and fixed with 4% paraformaldehyde for 30 min. Normal goat serum was used to block nonspecific binding sites. The coverslips were then incubated at 4°C overnight with antiaggrecan (1 : 200) and anticollagen II (1 : 200) antibody, followed by incubation with biotinylated IgG (1 : 400). The bound antibody was visualized using 3,3′-diaminobenzidine. The coverslips were mounted and examined under a microscope [11].

### 2.5. Semiquantitative Reverse Transcription-PCR (RT-PCR) Analysis

Total RNA was extracted from cells using TRIzol reagent (Shanghai Feijie Company, Shanghai, China). cDNA was reverse transcripted from total RNA using the First Strand cDNA Synthesis Kit (MBI Fermentas, Vilnius, Lithuania). PCR amplification was performed using the specific primers summarized in Table 1. PCR products were subjected to 1.5% agarose gel electrophoresis and stained with ethidium bromide. The bands were quantified by densitometry.

### 2.6. Statistical Analysis

Data are expressed as mean ± standard deviation. Differences among multiple groups were determined using one-way analysis of variance followed by Tukey's post hoc test. A value of *P* < 0.05 indicated statistical significance.

## 3. Results

### 3.1. Morphological Findings

The isolated cells attached to the culture plate within 24 h and showed a typical polygon shape. After culturing for 3–5 days, cells grew to confluence and could be subcultured.

### 3.2. Immunocytochemical Studies

**Figure 1:**
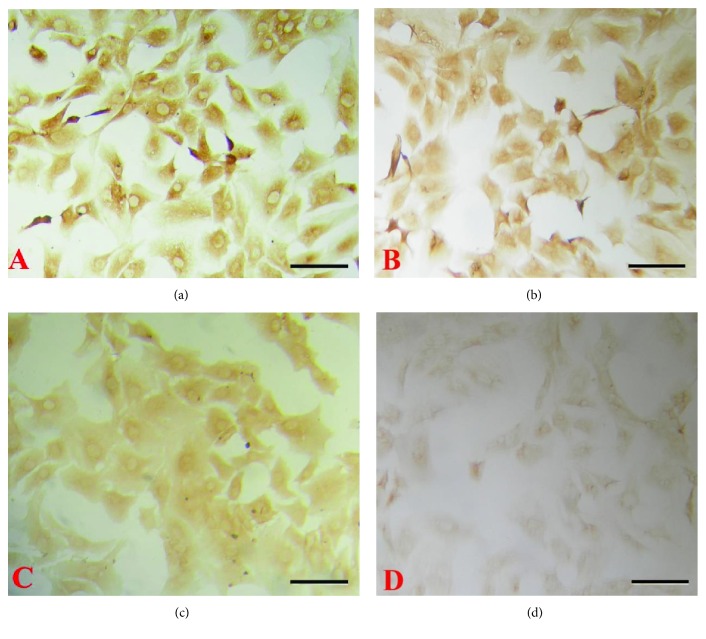
Effects of different concentrations of IL-1*β* on the expression of aggrecan in chondrocytes. Representative immunocytochemical images are shown. ((a) Control; (b) 1 ng/ml; (c) 10 ng/ml; (d) 100 ng/ml). Bar = 50 *μ*m.

**Figure 2:**
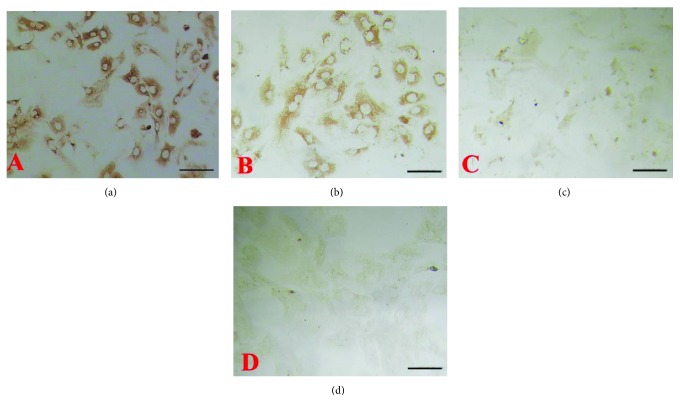
Effects of different concentrations of IL-1*β* on the expression of collagen II in chondrocytes. Representative immunocytochemical images are shown. ((a) Control; (b) 1 ng/ml; (c) 10 ng/ml; (d) 100 ng/ml). Bar = 50 *μ*m.

**Figure 3:**
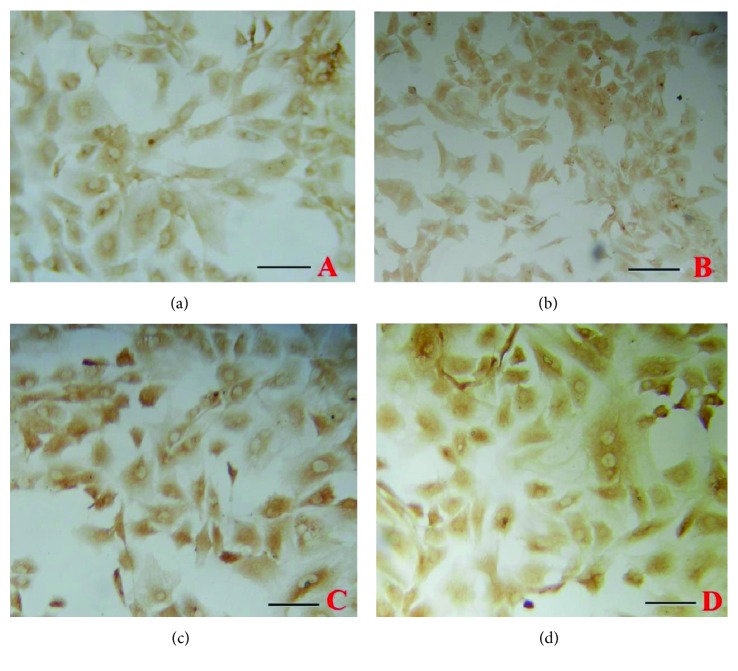
Effects of different concentrations of TGF-*β*1 on the expression of aggrecan in chondrocytes. Representative immunocytochemical images are shown. ((a) Control; (b) 1 ng/ml; (c) 10 ng/ml; (d) 100 ng/ml). Bar = 50 *μ*m.

**Figure 4:**
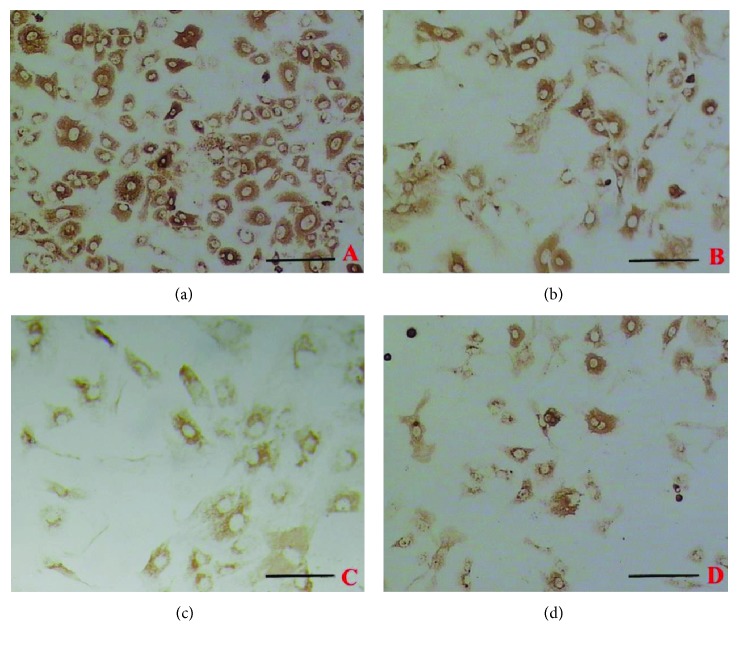
Effects of different concentrations of TGF-*β*1 on the expression of collagen II in chondrocytes. Representative immunocytochemical images are shown. ((a) Control; (b) 1 ng/ml; (c) 10 ng/ml; (d) 100 ng/ml). Bar = 50 *μ*m.

The amounts of aggrecan (Figure 1) and collagen II (Figure 2) were decreased with the increase in the concentration of IL-1*β* used. In contrast, treatment with high concentrations of TGF-*β*1 led to an increase in the aggrecan level (Figure 3) and decrease in the level of collagen II (Figure 4).

### 3.3. RT-PCR Analysis

**Figure 5:**
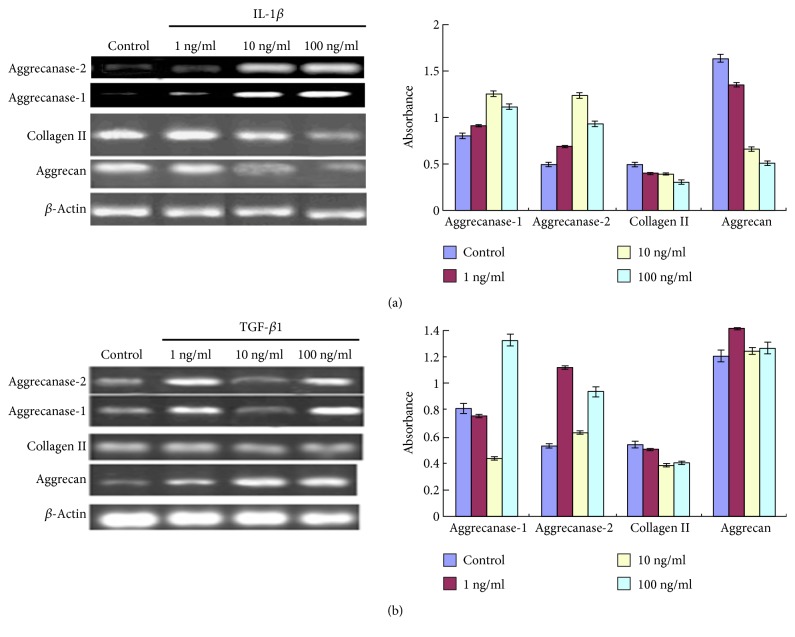
Analysis of the mRNA abundance of indicated genes in chondrocytes after treatment with different concentrations of IL-1*β* (a) and TGF-*β*1 (b).

Increased concentrations of IL-1*β* were associated with significantly (*P* < 0.05) lower levels of aggrecan and collagen II transcripts, compared to the control group (Figure 5(a)). IL-1*β* at the concentration of 10 ng/ml resulted in a maximal induction of aggrecanase-1 and aggrecanase-2 in chondrocytes. The aggrecan amounts increased after treatment with 1 and 10 ng/ml TGF-*β*1 but showed no significant change after treatment with 100 ng/ml TGF-*β*1 (Figure 5(b)). TGF-*β*1 treatment, especially at the concentration of 1 ng/ml, significantly induced the expression of aggrecanase-2. However, the level of aggrecanase-1 was significantly raised by the treatment with 100 ng/ml TGF-*β*1. Additionally, the collagen II level was not significant difference with different concentration of TGF-*β*1.

## 4. Discussion

Aggrecan and collagens are abundantly present in the extracellular matrix (ECM) of cartilaginous tissues [12]. Aggrecan is a proteoglycan that plays a critical role in chondrocyte growth and differentiation [13]. Aggrecanase-1 and/or aggrecanase-2 is the enzymes responsible for aggrecan cleavage during cytokine-induced cartilage degradation [14–16]. Aggrecanases have the ability to cleave aggrecan and are thus implicated in modulation of the behaviors of chondrocytes [17, 18]. Besides structural components, a number of growth factors such as IL-1*β*, TGF-*β*, bone morphogenetic protein (BMP), and fibroblast growth factor (FGF) are also detected in cartilage ECM. These growth factors affect multiple aspects of chondrocyte biology including cell proliferation, metabolism, and survival [19, 20]. IL-1*β* can suppress the proliferation of chondrocytes [21], while BMP and FGF are known to enhance chondrocyte growth [22]. However, the roles of TGF-*β* in chondrocytes remain controversial. It has been documented that TGF-*β* can facilitate protein synthesis and articular chondrocyte proliferation [23, 24], thus participating in the metabolism of cartilage ECM. De Haart et al. [25] reported that TGF-*β*1 is capable of stimulating the proliferation of primary chondrocytes but has a modest impact on the proliferation of chondrocytes after subculturing for several passages.

IL-1, consisting of two molecules (IL-1*α* and IL-1*β*), is primarily produced by mononuclear cells. IL-1*β* is an important component of cartilage ECM [26] and plays a negative role in chondrocyte proliferation [23]. Our data showed that IL-1*β* treatment significantly raised the amounts of aggrecanase-1 and aggrecanase-2 but decreased the production of aggrecan and collagen in chondrocytes. These results suggest that IL-1*β* can induce the expression of aggrecanases in chondrocytes and lead to ECM degradation, which in turn decreases the amount of collagen, consequently contributing to chondrocyte dedifferentiation. Our observations are consistent with a previous study [27].

Mature TGF-*β* (25 kDa in size), which is composed of two polypeptides linked with disulfide bonds, is a multifunctional cytokine. It plays a complex role in chondrocyte biology. It has been reported that TGF-*β*1 can stimulate protein synthesis and chondrocyte proliferation [26]. Similarly, Lee et al. [28] showed that TGF-*β*1-containing chitosan scaffolds provide proliferative advantages to chondrocytes, compared to control scaffolds. Shuler et al. [29] revealed that adenoviral delivery of TGF-*β*1 gene into chondrocytes led to a marked increase in collagen synthesis. However, TGF-*β*1 failed to rescue the collagen phenotype of dedifferentiated chondrocytes. Administration of high dose of TGF-*β*1 to damaged articular cartilage promoted tissue fibrosis [30]. In this study, we found that the collagen II level was reduced to the maximal extent after treatment with a moderate concentration of TGF-*β*1 (i.e., 10 ng/ml). In contrast, low to moderate concentrations of TGF-*β*1 decreased the expression of aggrecanase-1, while high concentration of TGF-*β*1 elevated the expression of aggrecanase-1 and aggrecanase-2. These results support the notion that TGF-*β*1 plays a dual role in chondrocyte differentiation [31].

Dedifferentiation is a major obstacle for chondrocyte-based tissue engineering. Both IL-1*β* and TGF-*β*1 can modulate the expression of aggrecan, collagen II, aggrecanase-1, and aggrecanase-2 in chondrocytes and thus have the potential to maintain chondrocyte phenotypes after serial passages.

## Figures and Tables

**Table 1:** PCR primers.

Gene	Primer sequences	Product size (bp)
*β*-actin	Sense 5′-GAGGGAAATCGTGCGTGAC-3′ Antisense 5′-TAGGAGCCAGGGCAGTAATCT-3′	353
Aggrecanase-1	Sense 5′-GCATCCGAAACCCTGTCAAC-3′ Antisense 5′-GGCGGTCAGCATCATAGTCC-3′	192
Aggrecanase-2	Sense 5′-AACTTGACATTTGGGCCTGA-3′ Antisense 5′-CAATGGCGGTAGGCAAACT-3′	290
Aggrecan	Sense 5′-GCTACACAGGTGAAGACTTTGTAGACATCC-3′ Antisense 5′-GCTGTGCCTCCTCAAATGTCAGAGAGTATCT-3′	478
Collagen II	Sense 5′-TGGTGCTGCTGACGCTGCTCATCGCCACGGTCCTA-3′ Antisense 5′-GCCTTCTGATCAAATCCTCCAGCCATCTGGGCCCGC-3′	339
